# NA-CONTROL: a study protocol for a randomised controlled trial to compare specific outpatient rehabilitation that targets cerebral mechanisms through relearning motor control and uses self-management strategies to improve functional capability of the upper extremity, to usual care in patients with neuralgic amyotrophy

**DOI:** 10.1186/s13063-019-3556-4

**Published:** 2019-08-07

**Authors:** Renee Lustenhouwer, Nens van Alfen, Ian G. M. Cameron, Ivan Toni, Alexander C. H. Geurts, Rick C. Helmich, Baziel G. M. van Engelen, Jan T. Groothuis

**Affiliations:** 1 Department of Rehabilitation, Donders Institute for Brain, Cognition and Behaviour, Radboud University Medical Center, P.O. Box 9101, 6500 HB Nijmegen, the Netherlands; 2 Donders Centre for Cognitive Neuroimaging, Donders Institute for Brain, Cognition and Behaviour, Radboud University, P.O. Box 9101, 6500 HB Nijmegen, the Netherlands; 3 Department of Neurology, Donders Institute for Brain, Cognition and Behaviour, Radboud University Medical Center, P.O. Box 9101, 6500 HB Nijmegen, the Netherlands

**Keywords:** Neuralgic amyotrophy, Parsonage Turner syndrome, Neurorehabilitation, Upper extremity, Scapular dyskinesia, Motor control, Peripheral nerve dysfunction, Maladaptive neuroplasticity, Physical therapy, Occupational therapy

## Abstract

**Background:**

Neuralgic amyotrophy (NA) is a distinct peripheral neurological disorder of the brachial plexus with a yearly incidence of 1/1000, which is characterised by acute severe upper extremity pain. Weakness of the stabilising shoulder muscles in the acute phase leads to compensatory strategies and abnormal motor control of the shoulder - scapular dyskinesia. Despite peripheral nerve recovery, scapular dyskinesia often persists, leading to debilitating residual complaints including pain and fatigue. Evidence suggests that persistent scapular dyskinesia in NA may result from maladaptive cerebral neuroplasticity, altering motor planning. Currently there is no proven effective causative treatment for the residual symptoms in NA. Moreover, the role of cerebral mechanisms in persistent scapular dyskinesia remains unclear.

**Methods:**

NA-CONTROL is a single-centre, randomised controlled trial comparing specific rehabilitation to usual care in NA. The rehabilitation programme combines relearning of motor control, targeting cerebral mechanisms, with self-management strategies. Fifty patients will be included. Patients are recruited through the Radboud university medical center Nijmegen, the Netherlands. Patients with a (suspected) diagnosis of NA, with lateralized symptoms and scapular dyskinesia in the right upper extremity, who are 18 years or older and not in the acute phase can be included. The primary outcome is the Shoulder Rating Questionnaire score, which measures functional capability of the upper extremity. Secondary clinical outcomes include measures of pain, fatigue, participation, reachable workspace, muscle strength and quality of life. In addition, motor planning is assessed with first-person motor imagery and functional magnetic resonance imaging. In a sub-study the patients are compared to 25 healthy participants, to determine the involvement of cerebral mechanisms. This will enable interpretation of cerebral changes associated with the rehabilitation programme and functional impairments in NA.

**Discussion:**

NA-CONTROL is the first randomised trial to investigate the effect of specific rehabilitation on residual complaints in NA. It also is the first study into the cerebral mechanisms that might underlie persistent scapular dyskinesia in NA. It thus may aid the further development of mechanism-based interventions for disturbed motor control in NA and in other peripheral neurological disorders.

**Trial registration:**

ClinicalTrials.gov, NCT03441347. Registered on 20 February 2018.

**Electronic supplementary material:**

The online version of this article (10.1186/s13063-019-3556-4) contains supplementary material, which is available to authorized users.

## Background

Plasticity is a feature of the central motor system that allows change in the system organisation. It enables the development of new motor strategies in a changing environment, which can be advantageous when learning a new skill, or when recovering from injury by adapting with a compensatory strategy. When elements of the motor system are damaged, for example in neurological disorders, neuroplasticity enables patients to regain (part of their) motor function [1, 2]. However, the plasticity is maladaptive when changes in the central motor system are not beneficial to functioning. Maladaptive neuroplasticity can have detrimental consequences, resulting in impaired motor function. There are indications that maladaptive motor strategies can occur not only in central, but also in peripheral nervous system disorders [3–6].

**Fig. 1:**
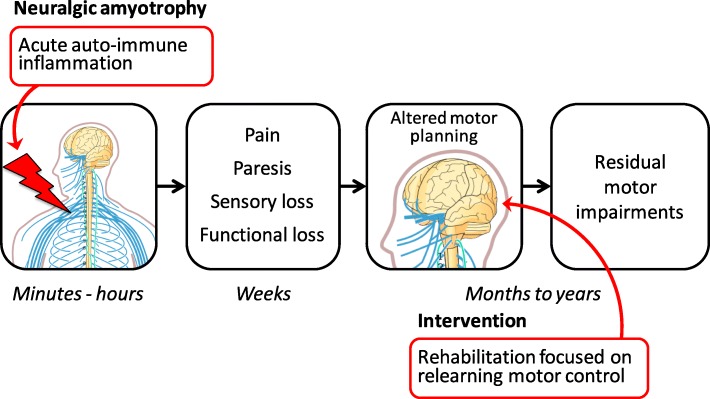
Cerebral reorganisation and rehabilitation after peripheral dysfunction in neuralgic amyotrophy. Schematic presentation of the concept that peripheral nerve damage leads to adaptations in motor planning that are compensatory in the acute phase, but lead to impaired motor control in the chronic phase. Neuralgic amyotrophy (NA) is an acute autoimmune inflammation of the brachial plexus, characterised by acute severe upper extremity pain and multifocal paresis. Many patients with NA develop abnormal motor control of the scapular region, scapular dyskinesia, which persists even after peripheral nerve recovery. This suggests that persistent scapular dyskinesia in NA may result from maladaptive neuroplasticity. Rehabilitation focused on relearning motor control, targeting cerebral mechanisms, can improve scapular movement and positioning, indicating that the impaired motor planning can be restored. This figure includes images that are adapted from *Nervous system diagram* licensed under the Creative Commons Attribution-Share Alike 4.0 International license, authored by Jordi March i Nogué and William Crochot

Neuralgic amyotrophy (NA) is a peripheral nervous system disorder in which (mal)adaptive central neuroplasticity might be important. There are several indications from clinical experience that (mal)adaptive central neuroplasticity is involved in this disorder [7]. However, little is currently known about the central mechanisms in this peripheral nervous system disorder. NA is a distinct peripheral nervous system disorder of the brachial plexus, with a yearly incidence ratio of 1/1000 [7, 8]. It can also be described as an asymmetric, autoimmune inflammation of the brachial plexus and peripheral nerves. In the acute phase, the inflammation causes damage to the affected nerves, leading to the characteristic acute severe upper extremity pain, multifocal paresis (i.e. muscle weakness) with functional impairments, and patchy areas of sensory loss. The long thoracic nerve that innervates the serratus anterior muscle, is affected in about 70% of patients with NA [9]. In a substantial subset of patients (> 50%), weakness of the serratus anterior muscle in the acute phase leads to compensatory, abnormal positioning and movement patterns of the scapula in the chronic phase. These patients develop chronic musculoskeletal pain in the paretic and compensating muscles, which leads to residual complaints of decreased functional capability of the affected upper extremity [10]. Many patients additionally suffer from impairment of activities of daily living, fatigue and decreased participation in daily occupations [7, 10]. The abnormal posture and movement patterns of the scapula are referred to as scapular dyskinesia. The concept of persisting shoulder complaints in NA is that through its plasticity, the motor system adapts to retain motor control of the shoulder region by forming compensatory movement patterns in the acute phase. After the acute phase, most of the damaged nerves recover over time and with this recovery, the strength of affected muscles, including the stabilising serratus anterior muscle, can return. However, recovery often does not lead to improved function because of dysfunctional coordination and instability of the scapula. Although some patients with NA recover well after 2–3 years, recovery is complicated in many [7, 9–12]. Residual complaints in NA are strongly correlated with persisting scapular dyskinesia [10]. There is currently no proven effective causative treatment for NA [13] and the usual care given, mostly standard physical therapy, is ineffective and may even worsen complaints in more than half of patients with NA [10]. The fact that scapular dyskinesia persists even when the peripheral nerves and strength of the stabilising scapula muscle recover implies that other, cerebral factors may play a role in explaining the residual symptoms and variable recovery in patients with NA. We introduce the concept that peripheral nerve damage in NA may lead to adaptations in motor planning and representations that are compensatory in the acute phase, but lead to impaired and dysfunctional motor control in the chronic phase (see Fig. 1).

This suggested involvement of maladaptive motor planning is illustrated by the promising results of specific rehabilitation after NA [14]. Through rehabilitation focused on relearning motor control, which thus targets cerebral mechanisms, patients with NA can relearn how to correctly position and move their shoulder and arm, which normalises scapular coordination and stability and improves functional capability of the upper extremity. A specific multidisciplinary and personalised rehabilitation programme, consisting of a visit to a specialised outpatient clinic that is followed by 8 sessions of physical and occupational therapy over a period of 16 weeks, has been developed at the Radboud University Medical Center (Radboudumc) in Nijmegen, the Netherlands. This programme combines relearning of motor control with self-management strategies. A clinical pilot study in eight participants showed that this rehabilitation programme can substantially relieve complaints and improve daily function at the level of activities, performance and participation. The programme was feasible, as all patients with NA were able to complete the entire programme. The number needed to treat was low, with 75% of the participants improving on the primary outcome measures [14].

Taken together, the available evidence strongly suggests that maladaptive motor planning plays a role in long-term symptoms and disability in NA. Indirect evidence includes the presence of persistent scapular dyskinesia despite peripheral nerve recovery and muscle strength, and the fact that rehabilitation focused on relearning motor control can normalise scapular movements and positioning. However, at this time, there is no direct evidence for (mal)adaptive cerebral neuroplasticity in NA. Despite the high incidence of NA (1/1000 a year) [8] and the presence of debilitating residual complaints [10], there are no randomised controlled trials (RCTs) investigating rehabilitation therapies targeting the residual complaints in NA. The NA-CONTROL study is an RCT designed to fill this gap; it compares the effect of a rehabilitation programme specifically designed for the residual complaints in this disorder to usual care in patients with NA. Additionally, the trial will combine clinical measures with measures derived to assess motor planning and representations in the central motor system, to provide mechanistic insights into how the rehabilitation programme could change central motor system plasticity.

## Objectives

### Primary objectives

The primary objective of this study is to determine the effect of a specific rehabilitation programme that combines relearning of motor control by targeting cerebral mechanisms with strategies to improve self-management, on functional capability of the upper extremity compared to usual care in patients with NA.

### Secondary objectives

#### The secondary objectives of this study are:

- To evaluate whether this rehabilitation programme results in improvements in a range of domains, including but not limited to scapular dyskinesia, participation, quality of life and personal factors such as pain and fatigue, compared to usual care in patients with NA.
- To assess the longer term (17 weeks post treatment) effects of the rehabilitation programme on a variety of outcomes, including but not limited to functional capability of the upper extremity, participation, quality of life and personal factors such as pain and fatigue.
- To determine the effect of this rehabilitation programme on cortical motor planning and representations compared to usual care in patients with NA.

## Methods

### Study description

The NA-CONTROL study is the first RCT to investigate treatment for residual complaints in neuralgic amyotrophy. It additionally investigates a relatively unexplored concept (i.e. the role of (mal)adaptive cerebral neuroplasticity in a disorder of the peripheral nervous system) and employs techniques (including functional magnetic resonance imaging (MRI)) that have not yet been used to study the underlying cerebral mechanisms in NA. This study is conducted at the Donders Institute for Brain Cognition and Behaviour and the departments of Rehabilitation and Neurology of the Radboudumc in Nijmegen, the Netherlands.

The effect of a rehabilitation programme on functional capability and motor planning of the upper extremity will be compared to that of usual care in this two-arm, single-centre, open-label RCT. The intervention group will receive a specific 17-week multidisciplinary rehabilitation programme at the Radboudumc outpatient clinic, focused on relearning motor control and self-management (see “Intervention” for more information). The usual care group will first receive usual care for a 17-week period, before entering the rehabilitation programme.

**Fig. 2:**
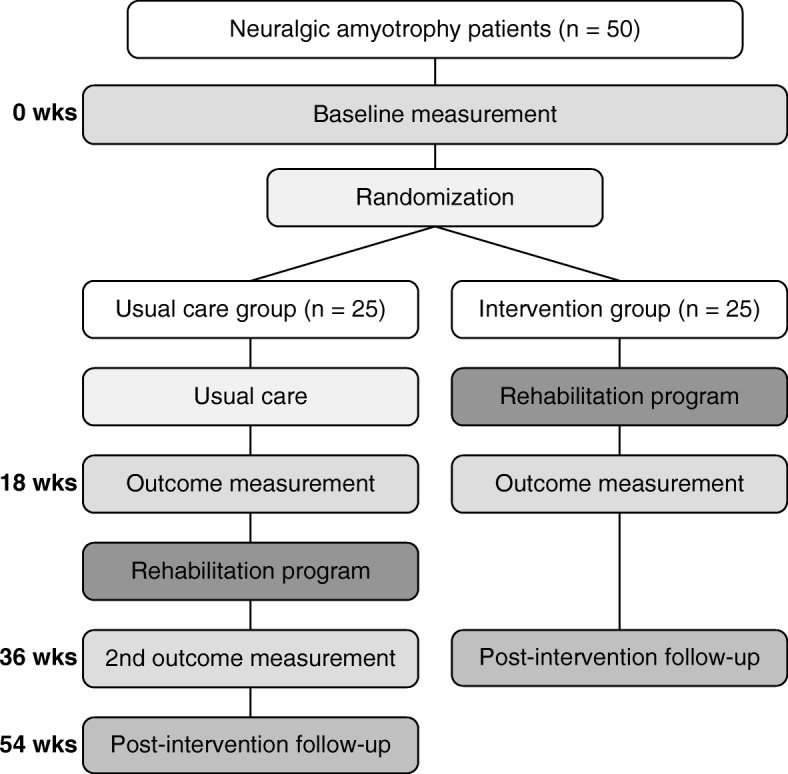
Flowchart of the study design: 50 neuralgic amyotrophy patients will be included. After the baseline measurement, participants are randomised into either the intervention group or the usual care group (1:1 ratio). After the first 17-week treatment period, both groups will undergo the first outcome measurement. The usual care group will then receive the 17-week rehabilitation program, after which they will undergo the second outcome measurement. Participants in both groups will complete a follow up from home 17 weeks after completing the rehabilitation program. Wks, weeks

Patients in both groups will be assessed in a single session at baseline. At the end of the baseline measurement, patients will be randomised into the intervention or usual care group (see “Randomisation” for more information). After the first 17 weeks of treatment (i.e. rehabilitation programme or usual care), both groups will be assessed in a second session (at 18 weeks post baseline). After this second assessment, the group that initially received usual care will then follow the specific rehabilitation programme. After completing the 17-week rehabilitation programme, the usual care group will be assessed a third time (at 36 weeks post baseline). All patients will be asked to fill out several questionnaires by e-mail 17 weeks after completing the rehabilitation programme. This follow up will be at either 36 weeks (intervention group) or 54 weeks (usual care group) post baseline. Figure 2 provides a flow chart of the study design.

#### Sub-study

The baseline measurements of all patients with NA who are assessed for the randomised controlled trial described in this publication (see Fig. 2) will be used for a sub-study. Patients with NA will be compared to 25 age-matched and sex-matched healthy controls in this sub-study. The healthy controls are assessed in a single session. The primary objective of this sub-study will be to determine if patients with NA have altered cerebral activity related to motor planning of their affected arm, compared to healthy controls and compared to their non-affected arm. This sub-study is important for the interpretation of the secondary objective (the effect of this rehabilitation programme on cortical motor planning and representations), to provide information about which cerebral changes from the rehabilitation programme are associated with functional impairments in NA.

### Study population

NA is more prevalent in men than in women, with an incidence ratio of 2:1, and can affect people of all ages. NA has an idiopathic form with a median age of onset around 40 years and a hereditary form with a median age of onset of about 28 years [7, 9]. Patients 18 years or older, with either form of NA can participate in the study.

#### Number of participants and sample size calculation

In total, 50 patients with NA will be recruited for participation in this study. The required sample size was calculated from the results from the pilot study on the effect of the rehabilitation programme [14]. The improvement in functional capability of the upper extremity measured with the Shoulder Rating Questionnaire, Dutch Language Version (SRQ-DLV) was used for this sample size calculation. With a conservative standardised effect size of 0.29 (improvement in SRQ-DLV [14]), power of 0.90 and two-tailed testing (α = 0.05), we calculated a required sample size of 42 patients. Assuming 20% dropout or non-compliance, we need to include 50 patients with NA.

The Radboudumc hosts the national referral centre and the only expert multidisciplinary outpatient clinic for patients with NA in the Netherlands. Each year around 400 new patients with NA are seen, most referred by Dutch neurologists or general physicians. Of these 400 patients, about 40% are estimated to be eligible for inclusion (see “Inclusion criteria”). We therefore expect to be able to recruit sufficient patients with NA for this study within the 2-year inclusion period.

#### Inclusion criteria

The treating rehabilitation physician or his/her physician assistant will judge whether a potential participant meets the inclusion criteria. In order to be eligible to participate in this study, a participant must meet all of the following criteria:

1. Diagnosis (suspected) of NA
  Initially, the (suspected) diagnosis of NA will be deduced from the information in the referral letter from the patient’s referring general physician or neurologist. If any uncertainty about the diagnosis remains, the rehabilitation physician or his/her physician assistant will contact the referring physician and/or patient. All patients will visit the specialised outpatient clinic, where the diagnosis will be either confirmed or discarded.
2. NA predominantly present in the right upper extremity
3. Being in the subacute or chronic phase of NA (i.e. no inflammation of the plexus, in practice, > 8 weeks after attack onset)
4. Presenting with scapular dyskinesia
5. Age ≥ 18 years
6. Right-hand dominance (as indicated with an Edinburgh Handedness Inventory (EHI) score > + 40)
7. Able to provide informed consent

#### Exclusion criteria

For the patients with NA, the treating rehabilitation physician or his/her physician assistant will judge whether a potential participant meets one or more of the exclusion criteria. A potential participant who meets any of the following criteria will be excluded:

1. Prior NA attacks of the lumbosacral plexus or the left upper extremity
2. Previous participation in the specific rehabilitation programme offered at the Radboudumc or the rehabilitation centre KINOS
3. Other neuromuscular disease affecting the shoulder girdle
4. Central nervous system disorder or neurological disorder (e.g. Parkinson disease, stroke etc.)
5. Pre-existing (bio)mechanical constraints of the shoulder girdle
6. A history of or recent periarticular fractures of the shoulder
7. Past surgery of the shoulder
8. Depressive mood disorder, as indicated by a score > 5 on the Beck Depression Inventory Fast Screen (BDI-FS)
9. Severe comorbidity
10. Ongoing participation in another scientific study that might interfere with the current study

Exclusion criteria for undergoing MRI are:

1. Pregnancy (current or planned within the study period)
2. The presence of metal parts that cannot be removed, in or on the upper body (including plates, screws, aneurysm clips, metal splinters, piercings, medical plasters or ossicle prosthesis but with the exception of dental fillings or crowns)
3. The presence of an electric implant (e.g. pacemaker, neurostimulator, insulin pump)
4. History of brain surgery
5. Claustrophobia
6. Epilepsy

#### Participant selection and enrolment

**Fig. 3:**
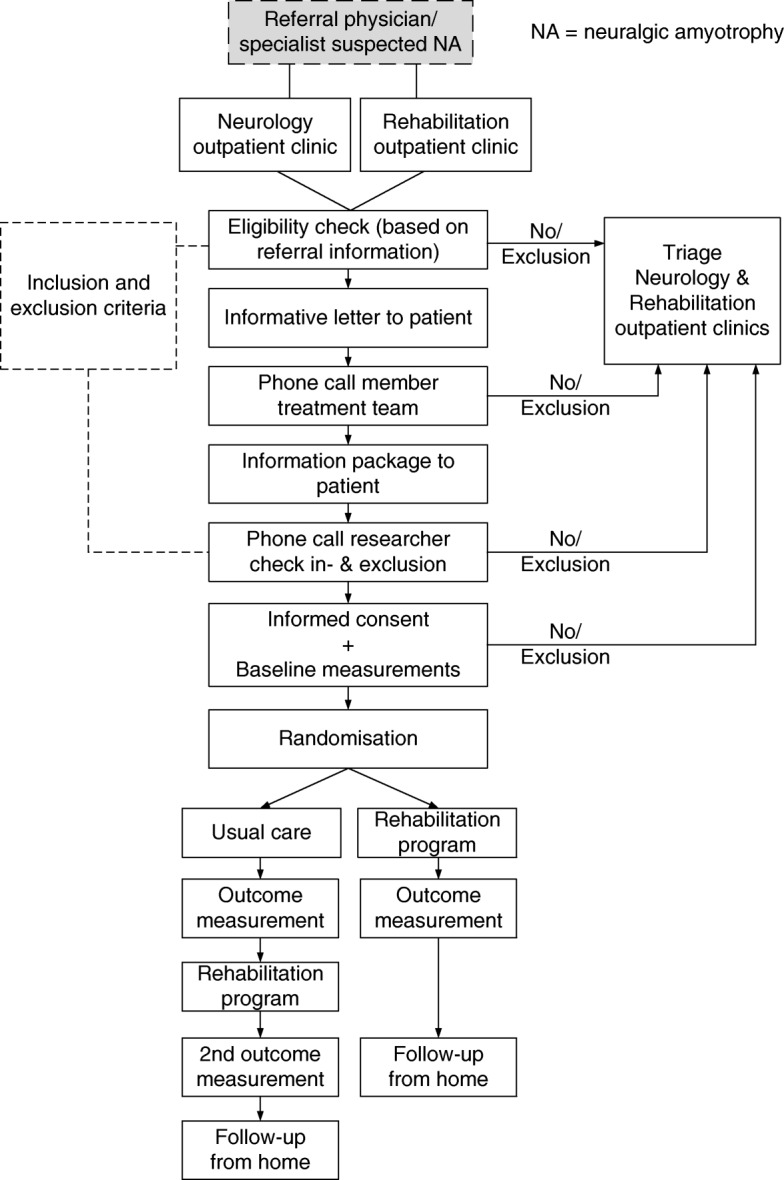
Flow-chart of patient recruitment, consent and other procedures of the study. NA, neuralgic amyotrophy

Figure 3 provides a flow-chart of the recruitment, consent and other procedures of patients in the NA-CONTROL study.

##### Identifying potential participants

All patients with NA that are newly referred to the Muscle Center of the Radboudumc during the study inclusion period will be checked for eligibility (i.e. meeting inclusion criteria 1–5) by a member of the treatment team through evaluation of the referral information. Eligible patients are informed about the NA-CONTROL study and their eligibility by post. At least a week after this notification letter is sent, a member of the treatment team will contact the patient to ask for his/her consent to be contacted by the coordinating researcher. Patients who express interest and provide consent will receive the extensive trial information package by e-mail.

##### Consenting participants

The coordinating researcher will contact patients who consent 7–14 days after the extensive trial information has been sent. After providing further clarification if needed, the coordinating researcher will state the inclusion and exclusion criteria. If the patient meets the inclusion and exclusion criteria, the researcher will ask the patient for oral consent and the baseline measurement will be scheduled.

Written informed consent will be obtained by the coordinating researcher at the research location. prior to the start of the baseline measurement. All participants will receive a copy of the signed informed consent form. The original signed informed consent forms will be kept at the study site. After written informed consent is obtained, the patient’s hand dominance will be determined using the Edinburgh handedness inventory (EHI) [15] and the patient will be screened for signs of depressive mood disorder using the Beck Depression Inventory-Fast Screen (see “Inclusion criteria”, “6” and “Exclusion criteria”, “8”, respectively).

Patients’ participation will be noted in their medical chart. As per national regulations, all participants’ general practitioners will be notified of their participation.

##### Ineligible and non-recruited patients

A patient’s decision to decline participation will in no way affect their treatment at the Radboudumc. This is clearly communicated to patients during all contacts. For patients who are not eligible, who express that they are not interested in participation or who do not meet all inclusion and exclusion criteria, the usual procedure is followed; they will be put on the regular waiting list for a consultation at our expert outpatient clinic. The content of the rehabilitation programme is the same for patients that participate in the trial as for those that receive the rehabilitation programme outside the study.

### Outcomes

See Table 1 for an overview of all outcome measures and their corresponding collection time points.

**Table 1:**
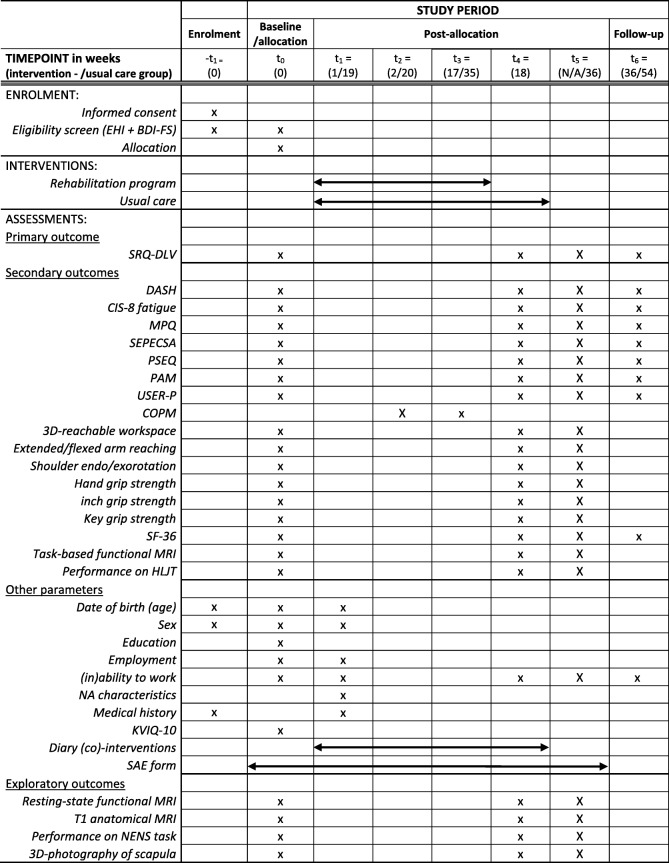
Standard protocol items: recommendation for interventional trials (SPIRIT) figure: schedule of enrolment, intervention and assessments during the trial. t_5_ is only applicable for patients in the usual care group. Patients in the usual care group start with the rehabilitation programme after the outcome measurement at t_4_. For this group, t_1_, t_2_ and t_3_ take place after t_4_. Abbreviations: 3D: 3 dimensional; BDI-FS: Beck depression inventory – fast screen; CIS-fatigue: checklist individual strength – fatigue; COPM: Canadian occupational performance measure; DASH: disability of arm, should and hand; EHI: Edinburgh handedness inventory; HLJT: hand laterality judgment task; KVIQ-10: kinesthetic and visual imagery questionnaire-10; MPQ: McGill pain questionnaire; MRI: magnetic resonance imaging; NA: neuralgic amyotrophy; NENS: neuromotor encoding in neuromuscular scapular dyskinesia; PAM: patient activation measure; PSEQ: pain self-efficacy questionnaire; SAE: serious adverse event; SEPECSA: self-efficacy for performing energy conservation strategies assessment; SF-36: short-form 36; SRQ-DLV: shoulder rating questionnaire – Dutch language version; USER-P: Utrecht scale for evaluation of rehabilitation – participation

#### Primary outcome

##### Functional capability of the upper extremity

The primary outcome measure for the clinical part of the RCT is the change in SRQ-DLV score from baseline to post intervention. The SRQ-DLV is a reliable and validated questionnaire measuring functional capability of the shoulder, arm and hand [16] and has been shown to be sensitive to (changes in) functional capability of the shoulder in patients with NA [14].

#### Secondary outcomes

Secondary outcome measures are divided into clinical measures and measures related to motor planning (see below).

##### Clinical

The secondary clinical outcome measures cover multiple domains of the International Classification of Functioning, Disability and Health [17]:

A. Activities and function: these will be assessed by administration of the following additional questionnaire
  1. Disability of Arm, Shoulder and Hand (DASH)
    The DASH questionnaire measures the functional capability of the affected upper extremity and has good clinimetric properties [18].
B. Personal factors: fatigue, pain, self-efficacy and patient activation are assessed using the following questionnaires:
  1. Checklist individual strength - subscale fatigue (CIS-fatigue)
    The CIS-fatigue measures experienced fatigue [19].

2. McGill Pain Questionnaire (MPQ)
  The MPQ measures the pain experienced. It assesses the nature, intensity, location, course and effect of the pain on daily life [20].
3. Self-efficacy for performing energy conservation strategies assessment (SEPECSA)
  The SEPECSA assesses how the patients perceive their ability to apply energy conservation strategies to their daily lives [21].
4. Pain self-efficacy questionnaire (PSEQ)
  The PSEQ assesses the confidence that people with ongoing pain have in performing activities while being in pain [22].

5. Patient activation measure (PAM)
  Patient’s activation with regard to their health and disease is assessed using the PAM. The PAM measures knowledge, skills and confidence in managing one’s own health and/or disease [23].

C. Participation: participation is assessed using the following measures:
  1. Utrecht scale for evaluation of rehabilitation-participation (USER-P)
    The USER-P is used to evaluate the effect of outpatient rehabilitation on participation [24].

2. Canadian Occupational Performance Measure (COPM)
  The COPM is used to evaluate occupational performance and satisfaction with performance of the most important daily occupations identified as problematic by the patient. It thus assesses occupational participation [25]. The COPM is administered during the first and last sessions of the specific rehabilitation programme [14]. These assessments will serve as a pre-intervention and post-intervention comparison within patients with NA who underwent the experimental intervention.

D. Body functions: within the body functions domain, we will assess the reachable workspace and several muscles/muscle groups
  1. 3D-reachable workspace
    Reachable workspace is an objective measure of upper extremity impairment [26]. It is quantified by the relative 3D surface area representing the portion of the unit hemisphere that is covered by the hand movements made during a standardised movement protocol [26]. The movement protocol covers cardinal movements of the shoulder and is performed in front of the Microsoft Kinect sensor-based reachable workspace analysis system [26].

2. The following strength measurements will be performed to determine maximal force exerted with several muscles/muscle groups on both sides (left and right upper extremity)
  The serratus anterior muscle measured using the MicroFET2®, digital manual muscle dynamometer, with the arm lifted to shoulder level, in the scapular plane while
    i. Reaching with the arm extended
    ii. Reaching with a flexed arm (elbow at 90°).
  Rotation of the shoulder measured using the MicroFET2®, digital manual muscle dynamometer, with the arm at 0° anteflexion, elbow flexed at 90° and thumb pointing upwards
    i. Endorotation
    ii. Exorotation.
  Hand grip measured using the Jamar® Hydraulic Hand dynamometer, with the arm at 0° anteflexion, elbow flexed at 90° and straight wrist.
  Pinch grip measured using the Baseline® LiTE Hydraulic Pinch Gauge with the arm at 0° anteflexion and elbow flexed at 90°. The pinch gauge is held between the index finger (top) and thumb.
  Key grip measured with Baseline® LiTE Hydraulic Pinch Gauge, with the arm at 0° anteflexion, elbow flexed at 90°. The pinch gauge is held between the thumb (top) and index finger (bottom).

E. Quality of life: quality of life will be assessed using the Short-Form 36 (SF-36)
  The SF-36 assesses experienced health and health-related quality of life [27].

##### Motor planning

Motor planning will be assessed using a motor imagery task during which functional MRI signal is recorded. Motor imagery involves mental simulation of a movement, without actual execution of that movement. It can be used as a tool to generate cortical motor states without movement production. As is evident by the presence of scapular dyskinesia, peripheral motor control is altered in NA. With motor imagery, changes in central motor control can be assessed, while controlling for alterations in peripheral factors. Empirical evidence shows that first-person motor imagery tasks are sensitive to motor control variables and use central neural mechanisms involved in action planning [28–30]:

A. Motor imagery task-based functional MRI

- Changes (as the result of rehabilitation and as the result of NA (assessed in the sub-study) in the neural mechanisms underlying motor planning and representations will be quantified by changes in the magnitudes of mean functional MRI signal, blood-oxygen-level-dependent (BOLD) activity associated with motor imagery during the Hand Laterality Judgment task (HLJT) (see “B”).
- Analyses of the functional MRI data will primarily be focused on the following brain regions: the extrastriate body area, the posterior parietal cortex in the intra-parietal sulcus region, and the precentral and postcentral gyri. These a priori regions of interest for analysis of functional MRI magnitude differences are chosen based on previous research using the same motor imagery task [1, 28, 30–32]. Additionally, we will employ a whole brain exploratory analysis of functional changes in NA outside these canonical motor imagery regions (see also “Exploratory outcomes”).
- Participants’ respiration will be recorded during the functional MRI scan to be able to control for noise introduced in the data.

B. Performance on the motor imagery task

- The HLJT [33] assesses central representations and planning of movements involving the upper extremity. Participants are asked to judge the laterality of line drawings of hands. The hands vary in laterality (left or right), view (palmar or dorsal) and degree of rotation (rotated − 135°, − 105°, − 75°, − 45°, 45°, 75°, 105°, 135° from the upright position). Participants are instructed to use their own hands as reference (i.e. imagine moving their upper extremity to match the hand shown on screen), without actually moving their upper extremity. As participants perform this task in the MRI scanner, they cannot rely on visual information to perform the task. The task consists of 32 blocks of 8 trials. The inter-trial interval ranges from 2000 to 3000 ms. Before each block, participants are instructed to place their hands in one of four positions: both hands with palms facing up, both hands palms facing down, one hand palm up (left/right) and one hand palm down (right/left). With this manipulation, participants use of first-person motor imagery can be checked through assessment of the posture effect: when using first person motor imagery, participants are faster for stimuli with a view that is congruent with the current posture of their own hand than for stimuli with a view that is incongruent with the posture of their hand [28]. First-person motor imagery use can further be corroborated by the effect of orientation: participants are faster for medially oriented stimuli that are associated with biomechanically easy movements towards the body midsagittal plane, than for laterally orientated stimuli, that are associated with biomechanically difficult movements away from the body midsagittal plane [28]. Participants are to report whether the hand drawing on display presented a left or right hand by pressing the corresponding button with their left or right foot.
- Behavioural performance on this task is evaluated by means of response times (i.e. time from stimulus onset to button press) and error rates (i.e. the number of incorrect trials divided by the total number of valid trials).
- During the task, alertness is monitored with an eye monitor and bipolar surface electromyography (EMG) over both thumbs is performed, to monitor muscle activity indicative of hand movements and exclude the influence of overt movements.

#### Other parameters

Some measures will be collected to describe the study population and if applicable, to correct for possible confounding effects:

A. Demographics: demographic data that will be collected includes age, sex and education level
B. NA characteristics: data on several characteristics of patients with NA will be collected. These include disease onset and duration, history of prior attacks and use of medication. Other characteristics may include, presence of atrophy, pattern of motor paresis, mechanical sensitivity of the plexus to pain, maximal severity of paresis during the attack and duration of primary pain.
C. Comorbidity: relevant information on comorbid conditions and medication use will be collected.
D. Motor imagery ability: there are individual differences in motor imagery ability [34]. As a subject’s ability to imagine movements might affect his/her performance on the motor imagery tasks in this study, motor imagery ability is assessed with the short version of the Kinesthetic and visual imagery questionnaire (KVIQ-10)*.* The KVIQ-10 assesses the clarity of the image (visual), and the intensity of the sensations (kinesthetic), that the subject is able to imagine from the first-person perspective [35].

#### Exploratory outcomes

In addition to its primary and secondary outcomes, the NA-CONTROL study includes some exploratory outcome measures that have not been used before to study NA or any similar patient populations. They were added to the study to gain additional insight in the underlying mechanisms of the residual complaints in this disorder.

##### Cerebral (re)organisation

Cerebral (re)organisation in NA will further be explored by means of additional MRI scans. For this purpose, functional and high-resolution anatomical images of the whole brain will be acquired on a 3 T Siemens whole body scanner at the Donders Centre for Cognitive Neuroimaging. An eye monitor will be utilised during the MRI scanning to monitor alertness. In addition to the secondary MRI outcome (i.e. the HLJT motor imagery task-based functional MRI signal), two different neuroimaging markers of cerebral organisation will be used:

A. Functional (re)organisation in rest
  Participants will be asked to lie still, think of nothing in particular and look at a fixation cross while 7 min of resting-state functional MRI data are obtained.
  Changes in functional activity and connectivity of several brain areas and cognitive resting-state networks (including the sensorimotor, fronto-parietal and executive control networks) will be analysed primarily with independent component analysis techniques on resting-state functional MRI data [36].

B. Structural (re)organization
  Changes in cerebral grey and white matter will be assessed using structural brain analysis based on anatomical T1-weighted MRI scans. We will perform voxel-based morphometry, a validated and fully automated technique for computational analysis of differences in global and local grey and/or white matter volume [37].
    As this part of the study is exploratory, analyses of the resting-state functional MRI data and structural MRI data will not be focused on a priori regions or networks of interests. In addition to the resting-state functional MRI and structural MRI scans, a new first-person motor imagery is employed to further assess motor planning and representations.

C. Neuromotor Encoding in Neuromuscular Scapular dyskinesia task (NENS-task)
  This first-person motor imagery task was specifically designed to investigate the impact of central motor planning in the clinical phenomenon of scapular dyskinesia. Participants are asked to imagine making pointing movements with their left or right elbow (novel movement) or finger (trained movements) towards targets shown on a computer screen, and to indicate with a right-foot button press when they have finished imaging the movement (i.e. when they have reached the target with the specified body part). Performance is evaluated by means of response times. Response time is defined as the time from stimulus onset to button press.
  For the NENS task, bipolar EMG of both serratus anterior muscles is used, to monitor muscle activity and exclude the influence of overt movements. The NENS task is performed outside the MRI environment, seated behind a computer screen.

##### Other exploratory outcomes

Position and orientation of the scapula: the 3D position and orientation of the scapula will be explored with a new measurement protocol. Anatomical locations for marker placement will be identified through manual palpation by a trained assessor (RL). These anatomical locations correspond to a thoracic reference plane (top and bottom of sternum, spine (vertebrae C7, T8), and three bony landmarks of the scapula (the angulus inferior, trigonum spinae and angulus acromialis) [38] at three levels of anteflexion (0°, 90° and 120°). Marker locations will be captured with 3D-photography at the Radboudumc’s 3D-photography laboratory. Subsequent analyses will provide the position and orientation of the scapula relative to the thoracic reference coordinate system.

### Randomisation and blinding

Patients with NA will be randomly assigned to either the experimental intervention or to the usual care in a 1:1 ratio. Patients are randomized using a Good Clinical Practice (GCP)-compliant system (Castor Electronic Data Capture), which employs stratified, variable block randomisation. To prevent uneven distribution of certain characteristics across groups, randomisation will be stratified according to two factors: sex (male/female) and age (4 blocks: 18–30, 30–42, 42–54, > 54 years). Randomisation will be performed on site (at the Donders Institute for Brain Cognition and Behaviour, Centre for Cognitive Neuroimaging) after the participant has completed the baseline measurement (see Table 1).

Due to the project design, it is not possible to blind participants or the assessor. Participants will inevitably know whether they are receiving the rehabilitation programme or usual care. The assessor cannot be blinded either, for multiple reasons: all assessments are performed by a single assessor; as the number of assessments differs across groups, the assessor will know which participant belongs to which group; as the rehabilitation programme takes place at the assessor’s workplace facility, the assessor could be un-blinded on encountering a participant visiting the facility for treatment.

### Withdrawal procedures

Participants can choose to withdraw their consent and leave the study at any time, without specifying the reason. No additional assessments will be obtained after a subject withdraws. Data that have been collected up until that point will be used for analyses. Participants have consented to this as part of the original informed consent. Patients that want to withdraw from the study can continue with the rehabilitation programme outside the trial if they wish to do so.

The investigator or a member of the treatment team can decide to withdraw a subject from the study for urgent medical reasons. Patients with NA who have a recurrent attack during their participation will be withdrawn from the study.

If a patient is withdrawn from the study before their visit to the expert outpatient clinic, they are asked to come to the Rehabilitation or Neurology outpatient clinic for a single interdisciplinary visit, to confirm their diagnosis of NA and provide treatment advice as usual.

### Patient retention

Patient retention is promoted in several ways. When participating in the specific rehabilitation programme, the patient is in regular contact with the Radboudumc treatment team (i.e. first five weekly visits, followed by two biweekly visits and two monthly visits over a 17-week period). Patients in the usual care group will be contacted at least twice during the 17-week control treatment period. The coordinating researcher will contact them by phone to inquire how the patient is and to remind them of the dairy in which they keep track of the care and/or treatment they have received. Both groups will be reminded about the post-treatment measurement(s) in the week prior to the upcoming visit.

### Intervention

The intervention under investigation is the rehabilitation programme developed and offered at the Radboudumc. This experimental intervention is compared to the usual care for NA in the Netherlands.

#### Experimental intervention

**Fig. 4:**
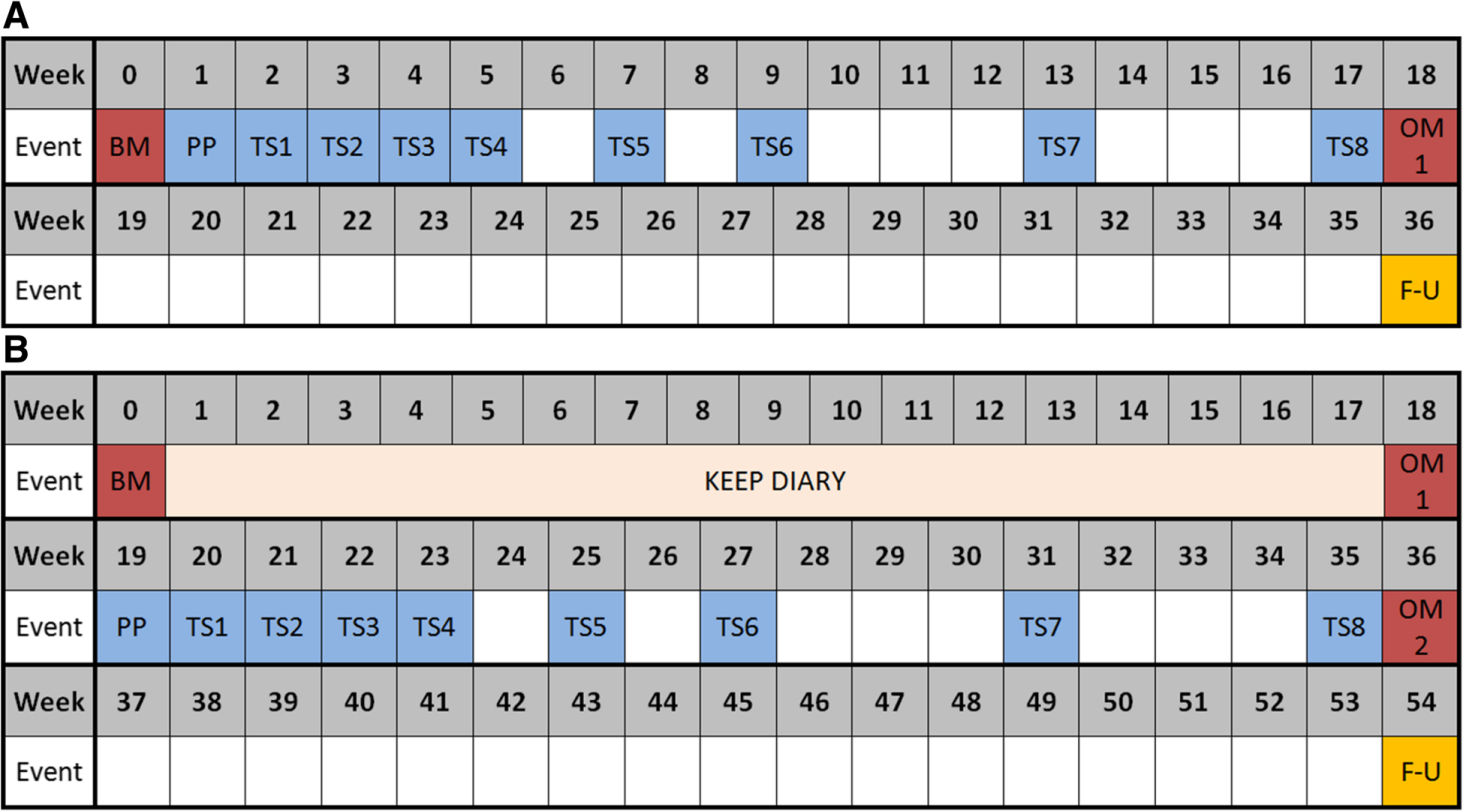
Overview of assessments and treatment for the intervention group (**a**) and the usual care group (**b**). A single treatment session consists of 1 h of physical therapy and 1 h of occupational therapy. BM, baseline measurement; PP, visit to out-patient plexus clinic; TS, treatment session; OM, outcome measurement; F-U, follow up

The experimental intervention is a 17-week specific rehabilitation programme. The programme starts with a visit to the specialised outpatient ‘Plexus clinic’ in week 1. During this visit, the patient is examined by a multidisciplinary team consisting of a rehabilitation physician, neurologist, physical therapist and occupational therapist. This specialised multidisciplinary team analyses the problems of the patient and provides a diagnosis and treatment advice. This treatment advice is implemented through four weekly sessions in weeks 2–5, two biweekly sessions in weeks 6–9 and two monthly sessions in weeks 10–17 (see Fig. 4 for an overview). Each of the eight treatment sessions involves one hour of occupational therapy and one hour of physical therapy. When needed, interdisciplinary strategies are employed.

**Fig. 5:**
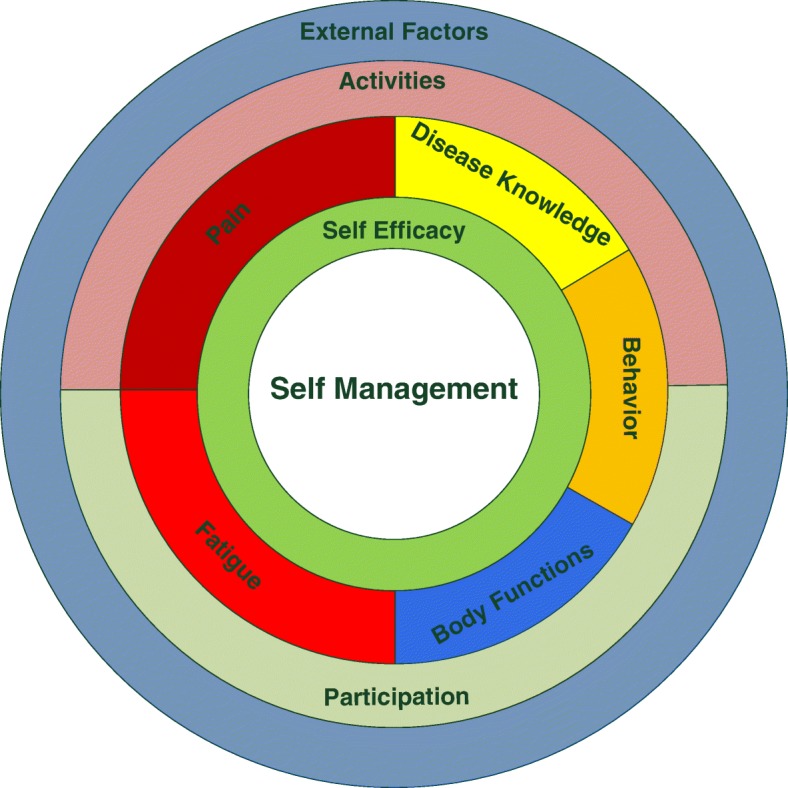
Treatment model with the components addressed during the rehabilitation programme. Issues in the outer two circles (External factors, Activity and Participation) form the main focus of the occupational therapy sessions. During the physical therapy sessions, the main focus is on improving body functions. All other components (i.e. disease knowledge, fatigue, pain, behaviour and self-efficacy and self-management) are addressed during occupational and physical therapy. This is accomplished through conveying knowledge of neuralgic amyotrophy and adaptation of behaviour related to functioning in daily life. Reproduced with permission from IJspeert et al. NeuroRehabilitation 2013;33:657–665 [14]

The model depicted in Fig. 5 forms the basis for the rehabilitation programme. This model consists of several components that are addressed during the intervention programme. The programme combines strategies of relearning motor control to normalise scapular stability and coordination [39], with strategies focused on self-management, including energy conservation [40], to enable daily occupations. The focus and extent to which components are addressed are adjusted to accommodate individual patients’ needs. For a full description of the rehabilitation programme, see IJspeert, Janssen [14] and van Eijk, Groothuis [7].

#### Usual care (control group)

The 25 patients with NA in the control group will receive usual care for 17 weeks. The usual care may vary for each individual, and may even consist of no treatment at all. Patients are asked to keep a diary during this period, in which they will report on the care/treatment they receive, including the type of care (e.g. physical or occupational therapy, acupuncture), number of sessions and composition of the care (e.g. strength training, aerobic training, massage). After 17 weeks, patients in the usual care group will start with the 17-week rehabilitation programme at the Radboudumc as described above.

#### Co-interventions

During the experimental intervention, co-interventions can be employed if needed. These co-interventions may include but are not limited to analgesics and steroid medication. While undergoing the experimental intervention, patients are asked to refrain from seeking additional treatment for the upper extremity outside the scope of the rehabilitation programme (e.g. additional therapy or care), unless this is discussed with the treating physician in advance. The use of any co-interventions will be registered.

Study participants are not allowed to participate in any other scientific study that might interfere with their participation in the NA-CONTROL study (from signing informed consent to completing the follow up from home).

#### Post-trial care

If deemed necessary by the treating clinician and desired by the patient, the treatment can be extended after the nine sessions that are part of the experimental intervention.

### Data collection and management

#### Data collection

Data will be collected as described under “Outcomes”. The assessor (the coordinating researcher) conducting the measurements is trained in collecting all measures that are obtained during the measurement protocol (i.e. operating MRI systems, applying bipolar EMG surface electrodes, conducting force measurements, palpating bony landmarks of the scapula and trunk, etc.).

#### Data management system

Most data are collected through laboratory-based measurement systems, or entered directly into the electronic case report form (CRF) in a GCP-compliant electronic data management system (Castor EDC, www.castoredc.com). This system utilises a log system with an automated audit trail. The Delegation of Responsibilities Log will identify all individuals responsible for data collection and handling, and management of the database.

A data management plan, detailing location of and access to study data and the code list, method of coding, back-up, locking and archiving of data, code list and analysis files has been submitted to and approved by the Board of Directors of the Radboudumc.

### Statistics and data analysis

#### Proposed analysis

All continuous variables will be summarised as the number of (missing) observations, mean, standard deviation, median and range. Categorical variables will be summarised as the number of (missing) observations and number and percentage in each category. This will be done separately for each time point (baseline, outcome measurement(s) and follow up) and for the change from baseline for each intervention group.

The primary clinical outcome (SRQ-DLV score) and most other clinical outcomes are linear or quasi-linear. Group differences in the effects of the intervention on primary and secondary outcome measures and the influence of possible effect modifiers will be investigated using generalised estimated equations analysis. If necessary, analyses will be adjusted for group differences in functional capability of the upper extremity (SRQ-DLV score), age and sex at baseline. Data will be analysed according to the intention-to-treat principle. Data will be appropriately transformed if necessary, to satisfy modelling assumptions. Prior to locking of the data, a statistical analysis plan will be drawn up and approved by a statistician and the principal investigator.

#### Missing data

Throughout data collection, measures will be taken where possible to minimise the occurrence of missing data. These measures include those mentioned under “Patient retention” and clear communication with individuals involved in data collection.

Once data collection is complete, the extent of missing data will be evaluated. Any patterns in missing data will be explored, especially in relation to the intervention groups. Missing data will be imputed if necessary and if possible.

### Monitoring

#### Data monitoring

As this study has a negligible risk classification it does not require a data monitoring committee. The study will be monitored by an independent, certified monitor according to the Netherlands Federation of University Medical Centres guidelines for monitoring of clinical studies. The frequency and extent of study monitoring is defined in a monitor plan that has been submitted to and approved by the Board of Directors of the Radboudumc.

#### (Serious) adverse events

All adverse events (AEs) reported spontaneously by the subject or observed by the investigator or the treatment team will be recorded. All AEs will be followed until they have abated or until a stable situation has been reached. Depending on the event, follow up may require additional tests or medical procedures as indicated and/or referral to the general physician or a medical specialist. All serious adverse events (SAEs) will be reported to the accredited medical ethical committee following national regulations.

### Good Clinical Practice

#### Ethical conduct of the study

The study will be conducted according to the principles of the Declaration of Helsinki (version 64th WMA General Assembly, Fortaleza, Brazil, October 2013) and in accordance with the Dutch Medical Research Involving Human Subjects Act (WMO). All personnel involved in the conduct of this study has received training on GCP. The principles of GCP will be followed throughout this study. This study protocol follows the Standard Protocol Items: Recommendations for Interventional Trials (SPIRIT) 2013 checklist (see Additional file 1).

#### Protocol amendments

All amendments will be notified to the accredited medical ethical committee. Non-substantial amendments will not be notified but will be recorded and filed by the investigator. Substantial amendments will not be implemented until approval of the accredited medical ethical committee has been obtained. Active participants will be informed and a new informed consent procedure will be started in the event of changes or additions to the protocol, which might influence participants’ decision to participate in the study.

#### Confidentiality

All clinical and research data collected for this study will be handled in such a way that participant confidentiality is ensured. All digital and hard copy records are kept in (digital) environments with limited access by appropriate staff only. Access rights and responsibilities are recorded on a designated list. Clinical information, images and research data will not be used by the study staff for any purposes other than the conduct of the study. Collection, sharing and maintenance of personal information during and after the study will comply with the international and national rules and regulations.

#### Study record retention

At the end of the study, all data will be checked and put into a validated database. Following Dutch national legislation, the database will be closed anonymously and stored in the Sponsor’s archive for 15 years.

#### Insurance and indemnity

The Sponsor has liability insurance and mandatory participants insurance for medical research involving human participants, which is in accordance with the legal requirements in the Netherlands (Article 7 WMO and the Measure regarding Compulsory lnsurance for Clinical Research in Humans of 2015).

### Reporting publications and notification of results

#### Scientific publications

Anonymised results will be published in national and international peer-reviewed journals.

#### Communication and dissemination

As the (inter)national expertise centre for NA, the Radboudumc is key in forming treatment guidelines and in the education of (peripheral) rehabilitation centres and primary care. This position enables rapid transfer of newly emerging knowledge on disease mechanisms and treatment obtained through this trial to the medical community. New treatment modalities, or adjusted or improved rehabilitation programmes can therefore be implemented rapidly.

Lay-friendly outcomes will be communicated to participants through trial newsletters and with the broader patient population through patient organisations. Where appropriate, information will be disseminated through newsletters, websites and at conferences in collaboration with patient organisations.

#### Authorship policy

Data arising from this study will be owned by the trial team and their employer. Authorship of publications coming from this study will follow the research code: publications are submitted only with authors who have made a substantial contribution to the research.

#### Peer review

The original proposal for the NA-CONTROL study has been reviewed by external reviewers appointed by the Prinses Beatrix Spierfonds, as part of the funding review process.

## Discussion

NA is a common peripheral nervous system disorder, which leaves many patients with persistent scapular dyskinesia and resulting residual symptoms that greatly impact quality of life for months to years after onset. To date, there are no clinical trials targeting these debilitating residual symptoms, and no explanation as to why these residual symptoms persist. The NA-CONTROL study is the first trial to evaluate a rehabilitation programme for residual symptoms after NA, that is focused on cerebral processes by relearning motor control and self-management strategies. This programme has been shown to increase functional capability of the upper extremity in a pilot study of patients with NA. The study design allows for comparison of the rehabilitation programme offered at the Radboudumc to usual care in patients with NA. Moreover, the lateralized nature of this disorder allows the use of the unaffected, contralateral upper extremity of patients with NA as an additional within-patient control method.

The NA-CONTROL study will investigate the effect of a specific rehabilitation programme on functional capability of the upper extremity. It thus aims to provide evidence for a tailored intervention to reduce residual complaints in NA. It additionally aims to provide insight into the cerebral mechanisms that might underlie the persistent motor problems in NA. This could confirm the clinical intuition that peripheral nervous system disorders (i.e. NA), may lead to maladaptive cerebral neuroplasticity, ultimately resulting in persistent symptoms, such as impaired motor control. Moreover, it will demonstrate whether such cerebral motor (mal)adaptations associated with scapular dyskinesia can be targeted by a specific rehabilitation programme. Knowledge on the extent of involvement of these cerebral mechanisms could help guide how much and which aspects of existing treatments should be focused on them. This knowledge may greatly aid in the further development of mechanism-based interventions for disturbed motor control in NA and in other peripheral neurological disorders. The NA-CONTROL study is an example of translational research as it combines clinical and non-clinical expertise and approaches.

## Trial status

This study is based on the protocol dated 5 December 2017 and has the following version numbers: NL63327.091.17–03 Algemeen Beoordelings-en Registratieformulier number (ABR-number) and 2017–3740 v3.0 Commissie Mensgebonden Onderzoek Arnhem-Nijmegen (medical ethical committee). Patient recruitment started in March 2018 and is ongoing at the time of submission of this study protocol. Recruitment is expected to be completed by January 2020.

## Additional file

Additional file 1: SPIRIT 2013 checklist: recommended items to address in a clinical trial protocol and related documents. (DOC 121 kb)

## Data Availability

Not applicable.

## Abbreviations

3D: 3 Dimensional
AEs: Adverse events
BDI-FS: Beck Depression Inventory - fast screen
BOLD: Blood-oxygen-level-dependent
CIS-fatigue: Checklist Individual Strength - fatigue
COPM: Canadian Occupational Performance Measure
DASH: Disability of Arm, Shoulder and Hand
EHI: Edinburgh Handedness Inventory
EMG: Electromyography
GCP: Good Clinical Practice
HLJT: Hand Laterality Judgment Task
KVIQ-10: Kinesthetic and Visual Imagery Questionnaire-10
MPQ: McGill Pain Questionnaire
MRI: Magnetic resonance imaging
NA: Neuralgic amyotrophy
NENS: Neuromotor encoding in neuromuscular scapular dyskinesia
PAM: Patient Activation Measure
PSEQ: Pain self-efficacy questionnaire
Radboudumc: Radboud University Medical Centre
RCT: Randomised controlled trial
SAEs: Serious adverse events
SEPECSA: Self-Efficacy for Performing Energy Conservation Strategies Assessment
SF-36: Short-Form 36
SRQ-DLV: Shoulder Rating Questionnaire - Dutch language version
USER-P: Utrecht Scale for Evaluation of Rehabilitation - participation
WMO: Dutch medical research involving human subjects act
